# Environmental Triggers of *lrgA* Expression in *Streptococcus mutans*

**DOI:** 10.3389/fmicb.2020.00018

**Published:** 2020-01-28

**Authors:** Ivan P. Ishkov, Sang-Joon Ahn, Kelly C. Rice, Stephen J. Hagen

**Affiliations:** ^1^Department of Physics, University of Florida, Gainesville, FL, United States; ^2^Department of Oral Biology, College of Dentistry, University of Florida, Gainesville, FL, United States; ^3^Department of Microbiology and Cell Science, Institute of Food and Agricultural Sciences, University of Florida, Gainesville, FL, United States

**Keywords:** *Streptococcus mutans*, bimodality, ccpA, fluorescence, catabolite repression, two-component systems, gamma distribution, pyruvate

## Abstract

The *cidAB* and *lrgAB* operons of *Streptococcus mutans* encode proteins that are structurally similar to the bacteriophage lambda family of holin-antiholin proteins, which are believed to facilitate cell death in other bacterial species. Although their precise function is not known, *cidAB* and *lrgAB* are linked to multiple virulence traits of *S. mutans*, including oxidative stress tolerance, biofilm formation, and autolysis. Here we investigate the regulation of *lrgAB* which in *S. mutans* shows a complex dependence on growth conditions that is not fully understood. By combining single-cell imaging of a fluorescent gene reporter with microfluidic control of the extracellular environment, we identify specific environmental cues that trigger *lrgA* expression and characterize cell-to-cell heterogeneity in *lrgA* activity. We find that the very abrupt activation of *lrgA* at stationary phase is tightly synchronized across the population. This activation is controlled by a small number of inputs that are sensitive to growth phase: extracellular pyruvate, glucose, and molecular oxygen. Activation of *lrgA* appears to be self-limiting, so that strong expression of *lrgA* is confined to a short interval of time. *lrgA* is programmed to switch on briefly at the end of exponential growth, as glucose and molecular oxygen are exhausted and extracellular pyruvate is available. Our findings are consistent with studies of other bacteria showing that homologs of *lrgAB* participate, with input from *lytST*, in the reimport of pyruvate for anaerobic fermentative growth.

## Introduction

The oral pathogen *Streptococcus mutans* (Loesche, 1986) possesses two operons, designated *cidAB* (SMU.1701/1700) and *lrgAB* (SMU.575/574) (Ahn et al., 2007), that are closely homologous to the *cidAB* and *lrgAB* operons which have been extensively studied in organisms such as *Bacillus subtilis* and *Staphylococcus aureus* (Brunskill and Bayles, 1996; Groicher et al., 2000; Rice et al., 2003; Rice and Bayles, 2003; Bayles, 2007; Charbonnier et al., 2017; van den Esker et al., 2017a,b). Sequence homology indicates that *cidAB* and *lrgAB* encode membrane proteins that are similar to holin-antiholin membrane proteins of the bacteriophage lambda family (Young, 1992, 2002; Young and Bläsi, 1995; Ahn et al., 2010). These proteins control autolysis and cell death by modulating the permeability of the bacterial cell wall (Young and Bläsi, 1995; Young, 2002; Pang et al., 2013; Saier and Reddy, 2015; van den Esker et al., 2017b). In *S. mutans*, deletions in *cidAB* or *lrgAB* have been shown to affect virulence-related behaviors such as autolysis, genetic competence, antibiotic resistance, biofilm development, and response to heat and oxidative stresses (Ahn et al., 2010, 2012, 2017; Ahn and Rice, 2016; Rice et al., 2017). Consequently, *cidAB* and *lrgAB* have been viewed as potentially encoding an *S. mutans* holin-antiholin system that responds to conditions of environmental stress by triggering autolysis and cell death (Chatfield et al., 2005; Ahn et al., 2010). However, the regulation of *cidAB* and *lrgAB* in *S. mutans* is complex and although the operons appear linked their precise function has not yet been established (Ahn et al., 2010, 2012; Ahn and Rice, 2016). Recent investigations of the link between these operons and cell death have found evidence that *lrgAB* homologs in *S. mutans* and other organisms may be more closely linked to metabolic control – and particularly to pyruvate utilization – than to cell death (Ahn et al., 2010; Charbonnier et al., 2017; van den Esker et al., 2017a,b; Kim et al., 2019). The *lrgAB* homolog in *B. subtilis*, which encodes a hetero-oligomeric membrane complex, was recently shown to function as a **p**yruvate **f**acilitated **t**ransporter and the operon was accordingly renamed *pftAB* (Charbonnier et al., 2017; van den Esker et al., 2017b). Also in *S. mutans*, *lrgAB* was very recently linked to the uptake of extracellular pyruvate in stationary phase (Ahn et al., 2019).

Expression of *S. mutans cidAB* and *lrgAB* responds to several two component signal transduction systems and to carbon catabolite repression, and these two operons display opposite patterns of expression during growth and maturation of a culture (Ahn et al., 2010, 2012; Ahn and Rice, 2016; Kim et al., 2019). The link to fluctuating parameters such as carbohydrate concentration and growth phase has made it difficult to identify specific cues that control the timing and extent of *cidAB* and *lrgAB* transcription. In addition, the kinetics and population heterogeneity of *S. mutans cidAB* and *lrgAB* expression have not been investigated. In this work, we use microfluidic and single-cell approaches to more precisely identify the extracellular cues that trigger *lrgAB.* We also characterize the temporal profile and cell-to-cell heterogeneity of *lrgAB* activity.

In *S. mutans*, the *cid* operon consists of *cidA* (342 bp) and *cidB* (696 bp), which overlap by four nucleotides (Ahn et al., 2010). The *lrg* operon includes *lrgA* (468 bp) and *lrgB* (732 bp) (Ahn et al., 2010). Both *cidAB* and *lrgAB* are sensitive to glucose availability, although the two operons behave oppositely. When *S. mutans* grows in limited glucose (less than 20 mM), *lrgAB* is not strongly expressed until the onset of stationary phase (Ahn et al., 2010; Kim et al., 2019). Higher initial glucose concentrations, exceeding 20 mM, reduce the stationary phase expression of *lrgAB*. By contrast, *cidAB* is robustly expressed during early growth in high glucose concentrations but is much less active later in growth or when initial glucose concentrations are less than about 20 mM (Ahn et al., 2010; Kim et al., 2019). Kim et al. have recently identified a catabolite responsive element (*cre*-site) region in the promoters of *cidAB* and *lrgAB*, indicating that the catabolite repression protein CcpA may enhance or suppress *cidAB* and *lrgAB* expression during early and late growth stages, respectively (Kim et al., 2019).

Several studies have found that *cidAB* and *lrgAB* respond to molecular oxygen and that deletions in either operon affect the ability of *S. mutans* to tolerate oxidative stress (Ahn et al., 2007, 2010). The Δ*cidAB* and Δ*lrgAB* deletion strains did not grow under aerobic conditions, although their anaerobic growth was reported similar to wild type (Ahn et al., 2010). Similarly, Δ*cidAB* and Δ*lrgAB* strains were unusually sensitive to superoxide anion (generated by paraquat) although not to hydroxyl radical (generated by hydrogen peroxide) (Ahn et al., 2010). Microarray experiments indicated that *lrgA* transcription increased in the presence of molecular oxygen during exponential growth phase (Ahn et al., 2007). A transcriptional profiling study found that *lrgAB* transcription at an optical density of 0.4 in a culture grown aerobically was 11-fold higher than in a culture grown in an anaerobic chamber (Ahn et al., 2007). Furthermore, *lrgA* and *lrgB* were also upregulated in thicker biofilms, perhaps suggesting sensitivity to oxygen conditions or other environmental stresses within the biofilm (Bayles, 2003, 2007, 2014; Shemesh et al., 2008).

The LytST two-component system also plays a role in *lrgAB* regulation in *S. mutans*, in which the *lytST* operon is located 175 nucleotides upstream of *lrgAB* (Ahn et al., 2010). LytST and its homologs have been closely linked to regulation of *lrgAB* homologs in many bacteria, including *Bacillus* and *Staphylococcus* species as well as *S. mutans* (Groicher et al., 2000; Kobayashi et al., 2001; Bayles, 2007; Ahn et al., 2010, 2012; van den Esker et al., 2017a). While *lrgAB* mRNA levels were observed to increase by 10^3^- to 10^4^-fold in late exponential phase of *S. mutans* under low glucose conditions (Ahn et al., 2010), the deletion of *lytST* or *lytS* reduced *lrgAB* expression throughout the growth curve. Deletion of *lytST* or *lytS* either eliminated (Ahn et al., 2010) or sharply suppressed (Ahn et al., 2012) the stationary phase rise in *lrgAB* mRNA levels (Ahn et al., 2010). This modulation of *lrgAB* induction by *lytS* was slightly greater at low oxygen conditions (Ahn et al., 2012), possibly indicating a link between LytST and environmental oxygen in regulation of *lrgAB.*

These prior findings show that growth-phase sensitive parameters such as glucose and oxygen interact to regulate *lrgAB* and may contribute to the suppression of *lrgAB* until the onset of stationary phase. Understanding this regulation in detail requires a greater degree of environmental control than is achieved through conventional, bulk culture methods. We have used microfluidics to maintain precise control of the environmental inputs that are suspected to influence *S. mutans lrgAB* and to explore the population profile and kinetics of *lrgAB* expression at the individual cell level. By imaging and quantifying activity of a green fluorescent protein reporter for the *lrgAB* promoter in individual *S. mutans* under controlled flow conditions, we were able to identify the environmental inputs that trigger activation of *lrgAB*.

## Methods

### Bacterial Strains, Plasmids, and Growth Conditions

Observing the effects of pyruvate and glucose on *lrgA* in *S. mutans* was possible through a *gfp* fusion to the promoter region of *lrgAB*, which was inserted into the pDL278 shuttle vector (carrying spectinomycin resistance) as described in Kim et al. (2019). The resulting plasmid was inserted into a wild-type UA159 and a *ccpA*-deficient mutant (Wen and Burne, 2002) to give the UA159/P*lrgA-gfp* and Δ*ccpA*/P*lrgA-gfp* strains, respectively (Kim et al., 2019). *S. mutans* with a *vicK* deletion is described in (Ahn and Burne, 2007).

The P*ldh-gfp* reporter strain was constructed by replacing the promoter region of our previous gfp reporter strains (Son et al., 2012, 2015). The P*ldh* region (about 200bp) was PCR-amplified with primers, incorporated HindIII and SpeI sites, respectively, and was cloned in front of the superfolder green fluorescent protein (sGFP) gene in the shuttle vector pDL278. The resulting construct was transformed into *S. mutans* wild-type UA159 strain.

The *lytST* overexpression strain (SAB163) was constructed using the method described in (Ahn and Rice, 2016). Briefly, a fragment containing the *ldh* promoter region (P*ldh*) and a polar kanamycin resistance gene (ΩKm-P*ldh*) was used to replace the *lytS* promoter region (P*lytS*): For construction of the ΩKm-P*ldh* cassette, a *ldh* promotor region (P*ldh*) was PCR-amplified from chromosomal DNA of *S. mutans* UA159 cell using primers P*ldh*-BamHI-FW and P*ldh*-SphI-RV and ligated to an ΩKm gene (digested with BamHI from pVT924) using BamHI site. For replacement of P*lytS*, two 0.5 kb fragments flanking the −35 and −10 regions of the *lytS* promoter were PCR-amplified using *lyt*S-A and *lyt*S-BamHI-B (left arm) and *lyt*S-SphI-C and *lyt*S-D (right arm) primers and ligated to the ΩKm-*Pldh* cassette using BamHI and SphI sites designed in each primer set. The final construct was then transformed into *S. mutans.* Testing *via* real-time qPCR confirmed about a 28-fold increase in *lytS* expression compared to the wild type. A *vicRK* overexpression strain (SAB164) was constructed using this same method. The primers used for overexpression strain construction and qPCR are listed in Table 1.

**Table 1:** Primers used in this study.

Primers used for construction of overexpression mutants		
Gene	Primer	Sequence
*ldh*	P*ldh*-BamHI-FW	AAA ACT CGT GGA TCC TTC ACT TGT T
	P*ldh*-SphI-RV	TGC AGT CAT GCA TGC AAC ATC TCC
	P*ldh*-HindIII-FW	CCG AAG CTT AAT AAC ACT CAT AGC
	P*ldh*-SpeI-RV	CAT ACT AGT AAC ATC TCC TTA TAA
*lytS*	*lytS*-A′	ACTGAACAGCCAGTGCACCA
	*lytS*-BamHI-B′	AAA GTT ACT GGA TCC ATT GCC ATG A
	*lytS*-SphI-C′	TAG GAG AAG GCA TGC ATG TTA ATG A
	*lytS*-D′	CAGTCAGACCAACGGCATCA
*vicR*	*vicR*-A′	CAGCAATAGCATCGGCCTTT
	*vicR*-BamHI-B′	TCT GAT TTT GGA TCC AAG CCC ACT T
	*vicR*-SphI-C′	AGC GAG GTA GCA TGC ATG AAG AAA A
	*vicR*-D′	GCGTCGCGTCAAAATGTACTC
**Primers used for qPCR**		
*lytS*	*lytS*-sense	TTGTCAGTTCTGCTTTGGTAGG
	*lytS*-antisense	CAATGACCTGCGAAGTAGATGG

*Restriction enzyme sites are underlined*.

For studies of P*lrgA* activation in a well plate system, overnight cultures of *S. mutans* UA159 and its derivatives were incubated in complex medium BHI at a temperature of 37°C in an atmosphere composed of 5% CO_2_. Antibiotics were used at the following concentrations where resistance is indicated in Table 2: erythromycin (10 μg ml^−1^), spectinomycin (1 mg ml^−1^), and kanamycin (1 mg ml^−1^). Overnight cultures were washed twice in phosphate buffered saline (PBS) of pH 7.2. They were then diluted 1:100 into defined medium [FMC (Terleckyj et al., 1975; De Furio et al., 2017)], pH corrected to 7.0 containing final concentrations of glucose and pyruvate dictated by the experiment conducted. Fresh cultures were allowed to grow to early exponential phase with an OD600 of 0.1 before being followed by any further testing. For single-cell studies as well as studies under a flow environment, overnight cultures of *S. mutans* were grown in BHI supplemented with an additional 20 mM glucose to ensure no activation of *lrgA*. Overnight cultures were washed twice in PBS and diluted 1:35 in fresh FMC before being allowed to incubate to an OD600 of 0.1.

**Table 2:** Strains and plasmids used.

Strain or plasmid	Genotypes and/or descriptions	Source or reference
***S. mutans* strains**
UA159	Wild-type	ATCC 700610
UA159/pDL278	Wild-type harboring an empty plasmid pDL278 Sp^r^	This study
Δ*lytST*	*lytST* genes replaced with a polar Km resistance cassette (ΩKm), Km^r^	Ahn et al. (2010)
Δ*vicK*	*vicK* gene replaced with non-polar Km resistance cassette, Km^r^	Ahn and Burne (2007)
SAB163	ΩKm-P*ldh* integrated into the chromosome of UA159 replacing P*lytS* (strain overexpressing *lytST*), Km^r^	This study
SAB164	ΩKm-P*ldh* integrated into the chromosome of UA159 replacing P*vicR* (strain overexpressing *vicRK*), Km^r^	This study
UA159/P*lrgA-gfp*	UA159 harboring P*lrgA-gfp* promoter fusion on pDL278, Sp^r^	Kim et al. (2019)
UA159/P*ldh-gfp*	UA159 harboring P*ldh-gfp* promoter fusion on pDL278, Sp^r^	This study
Δ*ccpA/*P*lrgA-gfp*	Δ*ccpA* harboring P*lrgA-gfp* promoter fusion on pDL278, Sp^r^	Kim et al. (2019)
Δ*vicK/*P*lrgA-gfp*	Δ*vicK* harboring P*lrgA-gfp* promoter fusion on pDL278, Sp^r^	This study
Δ*lytST/*P*lrgA-gfp*	Δ*lytST* harboring P*lrgA-gfp* promoter fusion on pDL278 Sp^r^	This study
SAB163/P*lrgA-gfp*	SAB163 harboring P*lrgA-gfp* promoter fusion on pDL278, Sp^r^	This study
SAB164/P*lrgA-gfp*	SAB164 harboring P*lrgA-gfp* promoter fusion on pDl278, Sp^r^	This study
**Plasmid**
pDL278	*E. coli – Streptococcus* shuttle vector, Sp^r^	LeBlanc et al. (1992)
pVT924	Vector harboring a ΩKm^r^ cassette	Y. Y. Chen, University of Florida

*Em, erythromycin; Sp, spectinomycin; Km; kanamycin*.

### Measuring Growth and Gene Activation in Bulk

**Figure 1:**
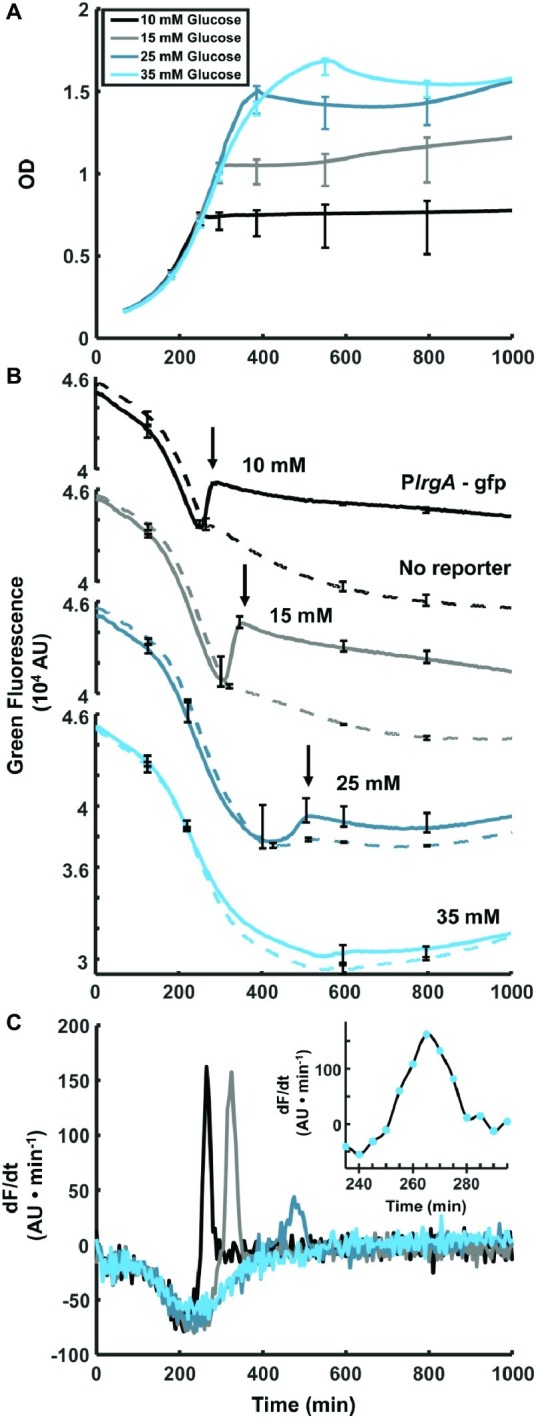
Observation of P*lrgA*-*gfp* fluorescence at the onset of stationary phase in *S. mutans*. **(A)** Optical density of P*lrgA-gfp* strain growing in defined medium at different initial glucose concentrations. **(B)** Green fluorescence of UA159/pDL278 (dashed curve) and P*lrgA-gfp* (solid curve) cultures is dominated by the steadily declining fluorescence of the medium, until about 250–300 min. Initial glucose concentrations are as indicated. The black arrows in (**B**) mark the abrupt burst of fluorescence in the P*lrgA-gfp* strain at the onset of stationary phase. **(C)** Comparison of the time derivatives of the green fluorescence for P*lrgA-gfp* shows that the burst of *lrgA* expression has a duration of 30–50 min. The inset in (**C**) shows the time derivative of reporter fluorescence in 10 mM glucose. The data shown in each panel represent one of three independent samples that were measured simultaneously. Error bars represent the standard deviation of the three independent samples.

**Figure 2:**
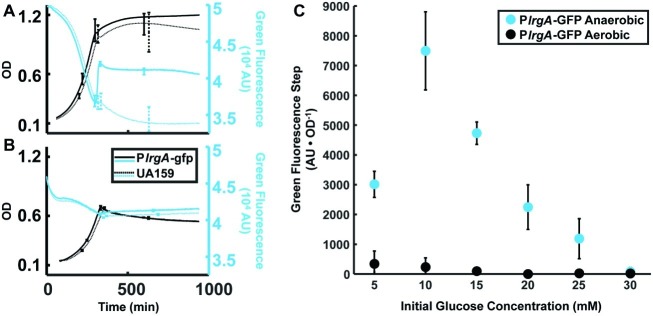
Effect of anaerobic **(A)** and aerobic **(B)** growth on the step increase in *lrgA* activity at stationary phase in static cultures of the *PlrgA-gfp* strain. OD (black curves) and green fluorescence (blue curves) are shown for the reporter (solid curve) and UA159/pDL278 (dashed curve) strains growing in medium supplemented with 15 mM glucose. Growth and fluorescence curves represent one of three independent samples that were measured simultaneously. Error bars represent the standard deviation of the three independent samples. Growth and fluorescence curves for all glucose concentrations used are included in Supplementary Figure S1. **(C)** Comparison of the step in *lrgA* activity for aerobic and anaerobic growth versus initial glucose concentration. The increase in *lrgA* activity in the reporter strain is measured as the magnitude of the fluorescence step (black arrows in Figure 1B) above background, normalized to the optical density. A small fluorescence step is detected in the aerobic cultures with low initial glucose concentration. Fluorescence steps in **(C)** represent the mean of three samples that were measured simultaneously. Error bars represent the corresponding standard deviations.

**Figure 3:**
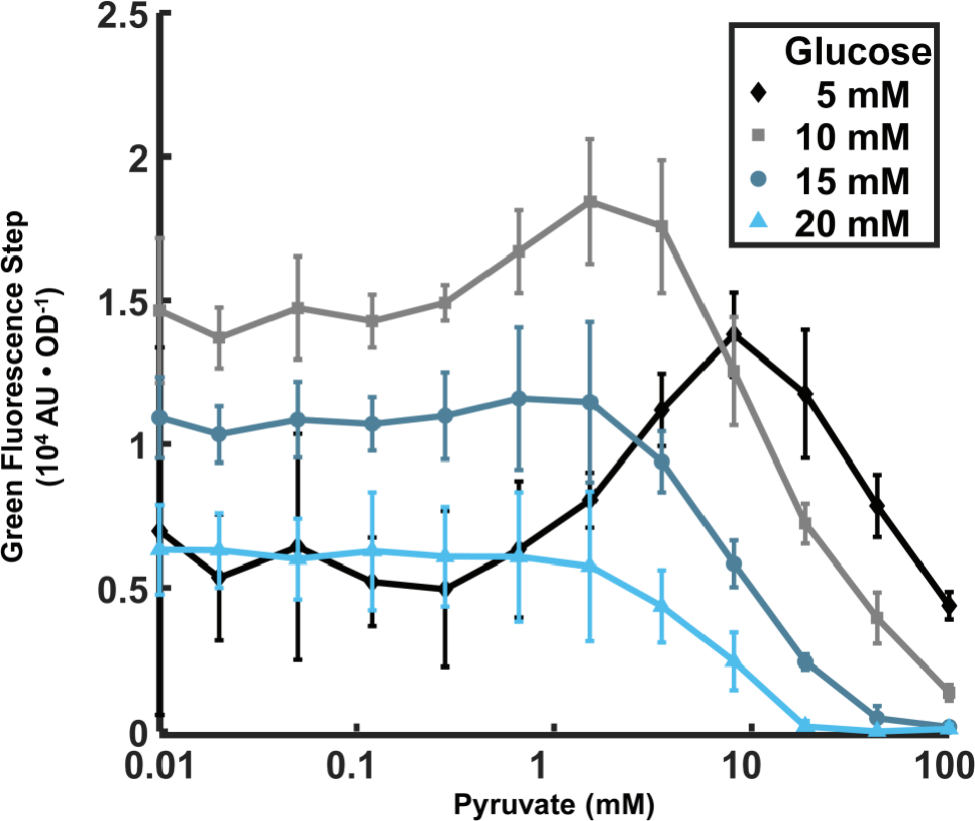
Dependence of *lrgA* expression step on initial pyruvate and glucose concentrations in cultures grown anaerobically. The increment in *PlrgA*-*gfp* reporter fluorescence (relative to baseline) at the onset of stationary phase is shown, normalized to the optical density. The data shown represent the mean of three independent samples. Error bars represent the corresponding standard deviations.

The data in Figures 1–3, 6 were collected with a BioTek Synergy 2 multimode wellplate reader. Overnight samples were first diluted 100-fold into fresh FMC media with the prepared initial carbohydrates necessary for the experiment. Samples were grown to an OD600 of 0.1 in prepared FMC media before being dispersed into 2 ml volumes (Figures 1, 2, 6) or 200 μl (Figure 3) on 24 or 96 well plates, respectively. Samples were overlaid with 410 μl mineral oil to facilitate anaerobic growth on a 24 well plate and 75 μl on a 96 well plate (Englander et al., 1987; Ahn et al., 2007, 2009, 2010; Ahn and Burne, 2007). Aerobic growth was facilitated with no mineral oil overlay and the plate was set to shake for 10 s every 2 min. Cultures grew in the well plates for 24–35 h and growth was monitored by optical density at 620 nm which was measured at 2 or 5 min intervals. Fluorescence was monitored by a green filter at 485–520 nm.

### Measuring *lrgA* Activation From Bulk

The fluorescence increase seen at the onset of stationary phase was calculated by the time derivative (slope) of the fluorescence curve obtained from a well plate reader and the time value at maximum slope. This time value corresponds to the inflection point of the fluorescence increase. An adjacent local minimum and maximum in the fluorescence are then found from the nearby time values at which the time derivative crosses zero. The difference between these maximum and minimum values is the fluorescence step at the onset of stationary phase. We then normalized this fluorescence step, dividing it by the optical density of the culture at its entry into stationary phase.

### Slide Experiments

Overnight cultures were diluted 1:35 fold into a 20 ml seed culture with a mineral oil overlay inside an incubator maintaining a 5% CO_2_ atmosphere at 37°C. About 3 μM propidium iodide (PI) was initially added to stain dead cells that had a weakened membrane integrity with a red fluorescent dye. To take phase and fluorescence images, a 600 μl sample was collected into a cuvette from the seed culture and an OD600 measurement was taken. The same sample was then ultra-sonicated to break up the cell chains and 4 μl deposited on a glass coverslip. Phase contrast and fluorescence images of the slide were taken on a Nikon TE2000U inverted microscope together with a Photometrics Prime camera and a green (or red for PI) filter set. Phase and fluorescence images were taken periodically throughout the full growth cycle of the culture until a stable, stationary phase optical density was reached. GFP concentration of individual cells was assessed from microscopy images using a method described previously (Kwak et al., 2012).

### Microfluidic Design

An Ibidi microfluidic slide (μ-slide VI, Ibidi USA) was used to measure activation levels of P*lrgA* under flow of medium at set rates. The microfluidic slide consisted of six flow channels that had dimensions of 0.1 mm × 1 mm × 17 mm for a total volume of 1.7 μl. Each of these rectangular channels allowed viewing through a microscope. Each channel had an inlet and an outlet that fit a standard Luer fitting which allowed the desired media to be pumped through the flow channels. The slide was secured to the stage of a Nikon TE2000U inverted microscope that is housed inside a temperature controlled Lexan chamber. While data were collected, the chamber was maintained at a constant 37°C by an electronic temperature controller (Son et al., 2015; De Furio et al., 2017; Underhill et al., 2018).

### Microfluidic Experiments

We cultured *S. mutans* P*lrgA-gfp* cells in defined (FMC) medium containing initially 10 mM glucose and grew them to 0.3–0.4 OD. We then sonicated the cells to break apart chains and loaded the cells into microfluidic flow channels. Cells were allowed to settle onto the lower window of the channel for 20 min, while the channel was mounted onto an inverted microscope in a temperature-controlled chamber. A flow of fresh medium was then supplied into the channels by a syringe pump at a rate of 1,000 μl/h for 30 min to replace and refresh the medium in the channels, connections, and fittings. After the 30 min purge, the pump rate was reduced to 20 μl/h and held constant for the duration of the experiment.

To ensure that the growth media for the microfluidic studies was sufficiently deoxygenated, we added an enzymatic oxygen scavenging system consisting of 2 U/ml glucose oxidase and 120 U/ml catalase (Englander et al., 1987). This system rapidly consumes O_2_ from the medium by breaking down glucose to yield gluconic acid and H_2_O as products. Although the glucose oxidase generates H_2_O_2_ as an intermediate product (which is then broken down by the catalase), *S. mutans* is tolerant of concentrations of H_2_O_2_ far higher than would be present during this reaction (De Furio et al., 2017). Formation of the mature GFP fluorophore does require molecular oxygen, and therefore, no GFP fluorescence is expected in the strict absence of oxygen (Cubitt et al., 1995). Oxygen concentrations in the range of about 5 μM, but not lower, appear sufficient for observation of fluorescence in improved GFP variants (Hansen et al., 2001; Iizuka et al., 2011). Our microfluidic system is not perfectly sealed and is somewhat permeable to atmospheric oxygen, and therefore, the glucose oxidase and catalase mixture is expected to reduce the steady state oxygen concentration to the range of 10–20 μM (Aitken et al., 2008; Baumann et al., 2008). Therefore, although the enzyme system drastically reduces molecular oxygen levels, it should allow sufficient oxygen to generate a GFP fluorescence signal (Vordermark et al., 2001; Wessel et al., 2014; Boudreau et al., 2016; Liu et al., 2017). In practice, we had no difficulty observing reporter-driven or constitutive production of GFP fluorescence in the deoxygenated growth media.

## Results

### A Burst of *lrgA* Activity Coincides With the Onset of Stationary Phase

To test our P*lrgA-gfp* fluorescent reporter strain and characterize *lrgA* expression in static cultures, we monitored the optical density and fluorescence of the reporter strain growing in well plates containing defined medium that was prepared with different initial concentrations of glucose. Figure 1A shows growth curves for the P*lrgA*-*gfp* reporting strain growing anaerobically under a layer of mineral oil (Englander et al., 1987; Ahn et al., 2007, 2009, 2010; Ahn and Burne, 2007). Figure 1B shows the green fluorescence (485 nm excitation, 528 nm emission) of *PlrgA-gfp*, relative to the UA159 (no reporter) background. For both strains, the growth medium contributes a large background fluorescence that declines steadily as the culture grows. In *PlrgA-gfp*, however, the green fluorescence increases abruptly as the culture enters stationary phase (arrows in Figure 1B), signaling a strong burst of *lrgA* expression. This rapid rise in green fluorescence is transient, as the green fluorescence gradually declines over longer time periods extending into stationary phase. The brief duration of the burst of *lrgA* expression is apparent from the time derivative of the fluorescence signal. Figure 1C shows that the fluorescent reporter for *lrgAB* is activated for no more than 30–50 min at the onset of stationary phase.

Figure 1B also shows that the initial glucose concentration of the medium influences the overall amount of *lrgA* expression that occurs during the burst. The size of the fluorescence rise in Figure 1B increases as the initial glucose is raised from 10 to 15 mM but declines as the initial glucose is further raised to 25 mM. At 35 mM initial glucose, the burst is not detected. These data are consistent with transcriptional data showing that *lrgAB* is upregulated 10^3^- to 10^4^-fold in late exponential phase, relative to early or mid-exponential phase (Ahn et al., 2010) and that very high initial glucose concentrations suppress this upregulation (Ahn et al., 2010; Kim et al., 2019).

### The Burst of *lrgA* Expression Is Observed Only Under Anaerobic Conditions

Prior studies have found interplay between *lrgAB* expression and molecular oxygen or oxidative stresses (Ahn and Burne, 2007; Deng et al., 2007; Ahn et al., 2010, 2012; Senadheera et al., 2012; Ahn and Rice, 2016). To more carefully assess the relationship between aerobic or anaerobic conditions and glucose availability on *lrgAB*, we measured the size of the stationary phase burst of reporter fluorescence in well plates that were growing anaerobically (with a mineral oil layer) or aerobically (open to air, with shaking), with different glucose concentrations. Figure 2 shows that, under anaerobic conditions, increasing the initial glucose to about 10 mM increases the amplitude of the *lrgA* expression burst. However, this amplitude falls monotonically if initial glucose is further increased. In P*lrgA-gfp* cultures grown aerobically, we observed a small burst of *lrgA* expression at initial glucose concentrations of 15 mM or less. No burst of *lrgA* expression was observed for initial glucose concentrations above 15 mM. Therefore, the burst of *lrgA* expression that occurs in a static culture requires anaerobic conditions as well as a moderately low initial glucose concentration. However, lower glucose concentration does not ensure higher *lrgA* expression; Figure 2C shows that the amplitude of the fluorescence burst declines at initial glucose concentrations lower than about 10 mM.

### Activation of *lrgAB* Requires the VicRK Two-Component System

The VicRK two-component system has previously been shown to alter *lrgAB* expression and linked to oxidative stress tolerance in *S. mutans* (Ahn and Burne, 2007; Deng et al., 2007; Senadheera et al., 2007; Ahn and Rice, 2016). To test whether the VicRK two-component system participates in regulation of *lrgAB*, we measured the activation of the P*lrgA-gfp* reporter in a strain (Δ*vicK*) carrying a deletion of *vicK*, which encodes the histidine kinase VicK, and in a *vicRK* overexpression strain (SAB164). Supplementary Figure S2 shows the growth and green fluorescence curves of these strains growing anaerobically and aerobically in 10 mM initial glucose. Under anaerobic conditions, P*lrgA* activity in the *vicK* deletion and overexpression strains is very similar to that observed in the wild type. Under aerobic condition, SAB164 showed a lack of P*lrgA* activity, like the wild type background. Unlike the wild type, however, the Δ*vicK* strain showed a modest but distinct burst of P*lrgA* activity at stationary phase in the aerobic medium. These data suggest that *vicK* has a repressing effect on *lrgA* under aerobic conditions.

### Extracellular Pyruvate Affects Stationary Phase Expression of *lrgA*

Recent findings that the LytST family of two component systems, which modulate the expression of *lrgA* homologs, can bind and sense external pyruvate (van den Esker et al., 2017a,b; Vilhena et al., 2018), and the observation that the pyruvate dehydrogenase complex in *S. mutans* is upregulated in late exponential phase (Kim et al., 2019), suggest that late growth expression of *lrgAB* in *S. mutans* may be connected to the presence of external pyruvate. We monitored the P*lrgA-gfp* reporter strain growing anaerobically in defined medium to which different concentrations of initial glucose and pyruvate were added. Figure 3 shows that very low concentrations of pyruvate (0–0.1 mM) had little effect on the magnitude of the step increase in GFP fluorescence at the onset of stationary phase, regardless of glucose concentration. However, further increases in pyruvate to 1.5–8 mM generally enhanced the stationary phase response of *lrgAB*, especially for cells growing at low glucose, 10 mM or less. Higher levels of pyruvate sharply reduced the activation of *lrgA*, until the fluorescence burst became undetectable at 100 mM pyruvate. These data show that initial glucose and pyruvate concentrations constitute a pair of external inputs that can modulate and maximize the stationary phase burst in *lrgA*, although both are inhibitory at higher concentrations.

### Expression of *lrgA* in Bulk Cultures at Stationary Phase Is Heterogeneous

**Figure 4:**
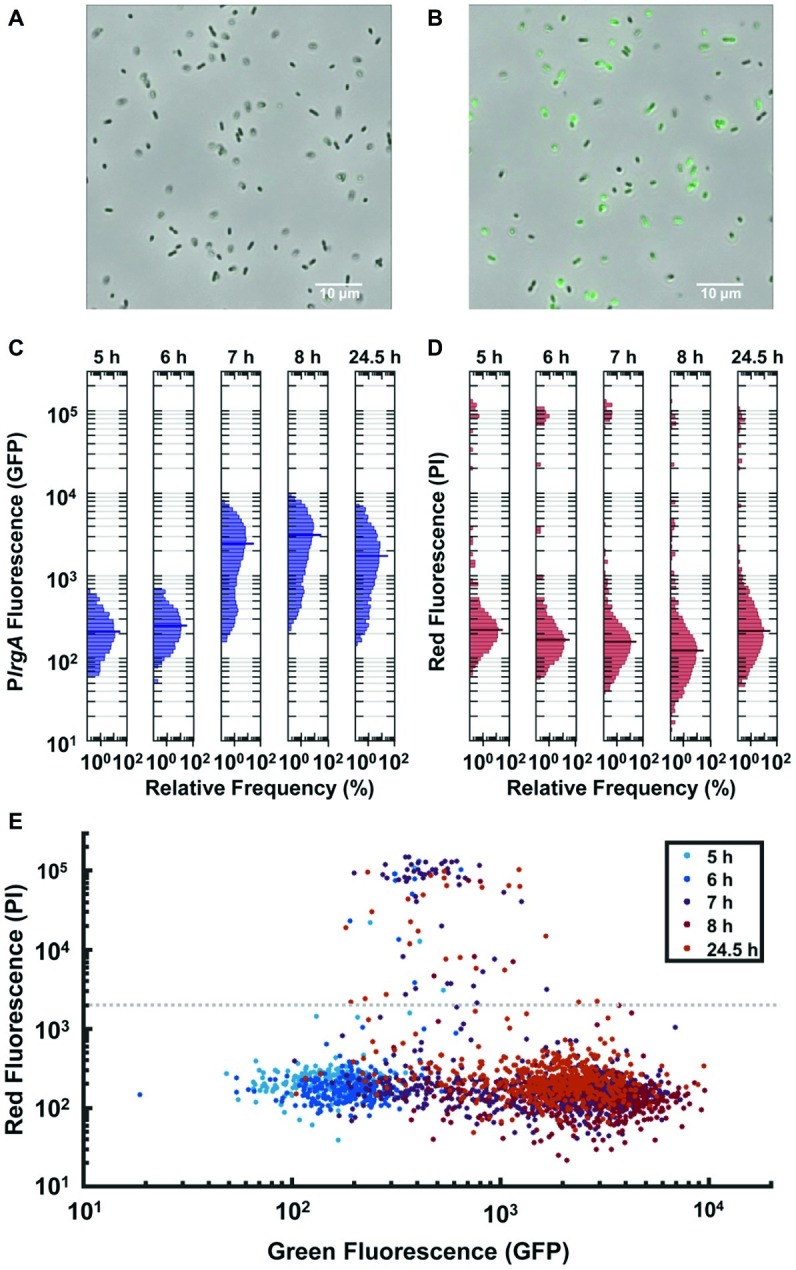
Observation of P*lrgA*-*gfp* reporter activity in individual cells extracted from bulk, anaerobic cultures grown in 10 mM initial glucose. Microscopy images of the reporter strain in phase-contrast (gray scale) are overlaid with GFP fluorescence (green) images at **(A)** 6 h and **(B)** 7 h of growth. **(C)** Histograms of individual cell GFP fluorescence measured at different times during growth. Fluorescence per cell is measured as described in (Kwak et al., 2012). **(D)** Histograms of propidium iodide (PI) fluorescence of the same individual cells as in **(C)**. The length of each horizontal bar indicates the percentage of cells that fluoresced at the indicated level. The heavy horizontal line in each histogram indicates the median fluorescence of the population. **(E)** Scatter plot comparing GFP and propidium iodide fluorescence of individual cells at different times during growth. Gray dashed line indicates a threshold for distinguishing PI-stained from non-PI-stained cells. Five individual images were collected and analyzed for each condition shown.

The very rapid burst of P*lrgA-gfp* fluorescence in Figure 1C shows that the timing of *lrgAB* activation is highly uniform in a population of cells. To test whether the degree of activation is equally homogeneous, we measured the fluorescence of individual P*lrgA-gfp* cells extracted from a static, bulk culture at different times during growth. We grew cultures anaerobically in defined medium prepared with 10 mM (initial) glucose, withdrew cells periodically, dispersed them on a glass slide, and imaged them in phase contrast and GFP fluorescence on an inverted microscope. Figure 4A shows that cells showed very little fluorescence through exponential phase, up through about 6 h. At 7 h, as the cells entered stationary phase, pronounced *lrgA* reporter fluorescence was observed (Figure 4B).

The GFP fluorescence after activation was highly variable from cell to cell, as shown by the histograms of individual cell GFP fluorescence in Figure 4C. While the histograms remain generally similar through exponential phase (roughly 5–6 h following inoculation), the heterogeneity in *lrgA* activation at 7 h is substantially greater. The median cell fluorescence at 7 h is roughly 10-fold greater than at 6 h, while the brightest cells at 7 h are roughly 11-fold brighter than the brightest cells at 6 h. The 7 h distribution has a slightly double-peaked (bimodal) character, suggesting that a subpopulation of cells have activated P*lrgA*, while other cells have not. The distribution shifts only slightly by 8 h or even 24 h, indicating that GFP concentrations in the population change little during stationary phase. This finding is consistent with Figures 1B,C, where the burst of *lrgA* expression lasts less than 1 h. Although the tight temporal synchrony of *lrgA* expression suggests that a single external cue triggers *lrgA* throughout the culture, the population variability in the resulting level of *lrg* expression indicates that not all cells in the static culture were immediately induced or that the *lrgAB* operon is not so tightly regulated as to enforce a consistent response among cells once induced.

To test whether the heterogenous reporter activity was due to the presence of dead cells, we monitored propidium iodide (PI) fluorescence of individual cells carrying the P*lrgA-gfp* reporter. Figure 4D shows the histograms of the red fluorescence of cells whose green fluorescence is shown in Figure 4C: The scatter plot of Figure 4E compares the green and red fluorescence of the same cells at different times during growth. Although the green fluorescence of the population generally trends upward as the culture reaches stationary phase, relatively few cells take up the PI stain. Of all cells measured, only 3.9% show red fluorescence above the threshold that is defined by the gray dashed line in Figure 4E. Because the PI staining shows little indication of compromised cells, the heterogenous response of *lrgA* in Figure 4C likely indicates that as a bulk culture enters stationary phase many intact cells simply fail to activate *lrgA*.

### Activation of *lrgA* in Controlled Flow Requires Pyruvate and Deoxygenation

High initial glucose concentrations suppress the activation of *lrgAB* at the onset of stationary phase. This finding suggests that the *lrgAB* expression burst may be triggered by the exhaustion of glucose from the growth medium and the alleviation of catabolite repression of *lrgAB*. However, Figures 2, 3 also show a role for molecular oxygen, possibly in combination with extracellular pyruvate. A difficulty with using bulk, static cultures to study these inputs is that they are altered by the growth and maturation of the culture and are poorly defined once a static culture has grown to stationary phase. To identify more precisely the factors that trigger *lrgAB*, we used microfluidic flow devices to apply a stable flow of fresh, defined medium to cells that were under continuous observation. We loaded P*lrgA-gfp* cells into microfluidic flow chambers (section “Methods”) on a microscope stage and supplied a continuous flow of fresh, defined medium through each channel. The flow rate of 20 μl/h ensured that the 1.7 μl volume of medium within each channel was replaced every 5.1 min. This flow prevented the cells adhered in the channels from modifying their chemical environment.

**Figure 5:**
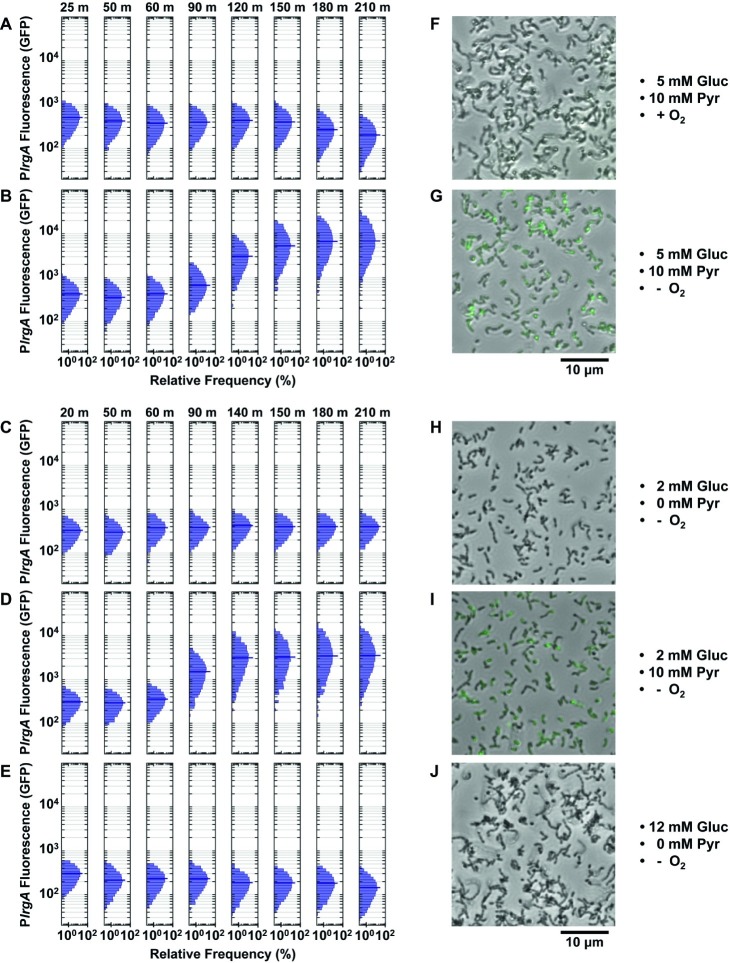
Effect of O_2_, glucose, and pyruvate on a P*lrgA-gfp* reporter in microfluidic flow. The histograms show the green fluorescence of individual cells adhered within microfluidic channels and subject to a steady flow of fresh, defined medium: **(A)** aerobic medium containing 5 mM glucose/10 mM pyruvate; **(B)** anoxic medium containing 5 mM glucose/10 mM pyruvate; **(C)** anoxic medium containing 2 mM glucose (no added pyruvate); **(D)** anoxic medium containing 2 mM glucose/10 mM pyruvate; **(E)** anoxic medium containing 12 mM glucose (no added pyruvate); **(F–J)** Phase microscopy images (collected at 150 min) of the reporter strain are shown in gray scale, overlaid with GFP fluorescence (green) images. Five individual images were collected and analyzed for each condition shown.

Figure 5A shows the response of cells that were provided an air-equilibrated (aerobic) defined medium containing 5 mM glucose and 10 mM pyruvate. Expression of *lrgAB* remained at basal levels, similar to the fluorescence of the UA159 (no reporter) strain (Supplementary Figure S3A). (A modest decline in the average fluorescence at 180 and 210 min is an artifact of rampant growth affecting the image analysis algorithm). Similar flow experiments using medium that was either fully aerated or partially deoxygenated by stirring in vacuum or under N_2_ produced GFP histograms very similar to Figure 5A (data not shown): No activation of *lrgA* was observed in flow experiments at any combination of glucose and/or pyruvate concentrations when the supplied media were aerobic or partially deoxygenated.

We therefore tested whether more rigorous deoxygenation was needed to mimic the conditions of a static, anaerobic (mineral oil layer) well plate and induce a response from *lrgAB*. Figures 5B,D show the results when the growth medium was made more stringently anoxic by the addition of an enzymatic system that scavenges molecular oxygen (“Methods” section). These anoxic media induced robust expression of *lrgAB*. Strong GFP production was observed after 90–120 min of flow of anoxic medium that contained 5 mM glucose and 10 mM pyruvate (Figure 5B) or 2 mM glucose/10 mM pyruvate (Figure 5D). The first 50 min of the 90–120 min delay is attributable to replacement of partially deoxygenated medium that was initially present in the flow connections.

By contrast a strain carrying *gfp* under control of a constitutive promoter (P*ldh*) was strongly activated under both aerobic and anaerobic conditions, as shown in Supplementary Figures S3B,C.

Our data therefore demonstrate that rigorous deoxygenation is a condition for the *lrgAB* reporter to activate in a continuous flow experiment. We then tested whether pyruvate was also required. Deoxygenated medium containing 2 (Figure 5C) or 12 mM (Figure 5E) glucose, without added pyruvate, did not activate *lrgA*.

In summary, strong upregulation of *lrgA* was only achieved under continuous flow conditions when the supplied medium was rigorously deoxygenated and contained added pyruvate. Once these conditions were present, the concentration of glucose (over the range 2–5 mM) had only modest additional effect on *lrgAB* activity. Microscopy images in Figures 5G,I show cells with an activated *lrgAB* reporter (green) after 150 min in supplied medium, distinctly brighter than cells growing in aerobic or non-pyruvate media (Figures 5F,H,J).

The activation of *lrgAB* in the flow conditions of Figures 5B,D was also more narrowly distributed than in the static medium study of Figure 4C. The 210 min histograms in Figures 5B,D lack the broad, heterogeneous *lrgAB* expression that is seen in the activated (7 h) cells in Figure 4C and suggest that the more homogeneous chemical environment of the flow conditions leads to a more uniform *lrgAB* response of the population.

### Deletion of *ccpA* Does Not Eliminate Burst Expression of *lrgA*

**Figure 6:**
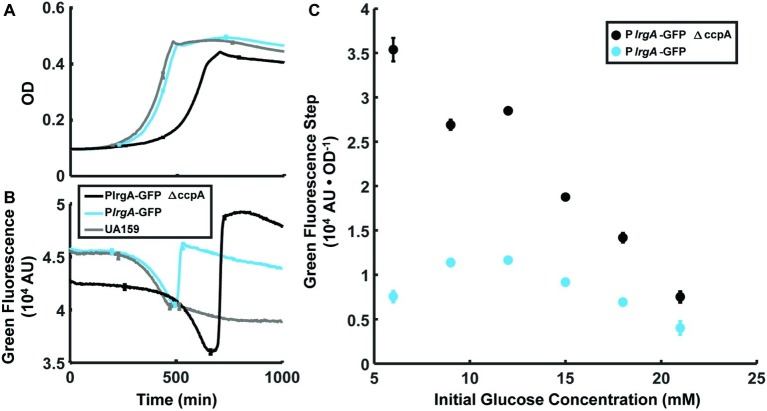
Effect of *ccpA* on the *lrgA* expression burst at stationary phase in static grown cultures. Growth **(A)** and *PlrgA-gfp* reporter fluorescence **(B)** of UA159 background (blue) and Δ*ccpA* (black) growing anaerobically in 12 mM initial glucose. Growth and fluorescence curves represent one of three independent samples that were measured simultaneously. Error bars represent the standard deviation of the three independent samples. **(C)** Size of the fluorescence activation step at stationary phase, normalized to optical density. Fluorescence steps represent the mean of three samples that were measured simultaneously. Error bars represent the corresponding standard deviations.

The above data strongly suggest that molecular oxygen and glucose both inhibit *lrgA* activation until the conclusion of exponential growth. Because a *cre*-site for the catabolite repressor protein CcpA was recently identified (Kim et al., 2019) in the *lrgA* promoter region, we investigated a possible role for CcpA in suppressing *lrgAB* activity. We compared expression of a P*lrgA-gfp* reporter in the wild type background and in a Δ*ccpA* strain, both growing anaerobically, for a range of glucose concentrations. Figures 6A,B show a similar abrupt onset of *lrgAB* expression at the beginning of stationary phase in the *ccpA* deletion. In Figure 6C the amplitude of the expression step is larger in the *ccpA* deletion than in UA159 background, where the relative effect is larger at low initial glucose levels. Therefore, although catabolite repression may partially inhibit the magnitude of the expression burst, it evidently does not control the timing of the burst.

### Overexpression of *lytST* Permits *lrgA* Expression in Aerobic Media

The LytST two-component system is implicated in the regulation of *lrgAB* homologs, as for example, in *B. subtilis* where *lytST* was linked to pyruvate sensing and shown to be required for expression of the *lrgA* homolog (van den Esker et al., 2017a). Prior studies of *S. mutans* in static, bulk cultures showed that deletion of *lytS* (Ahn et al., 2012) or *lytST* (Ahn et al., 2010) abolished the stationary phase expression of *lrgAB*. Supplementary Figure S4 shows growth and fluorescence curves of strains lacking (Δ*lytST*) or overexpressing (SAB163) *lytST*, growing in 10 mM glucose. The *lytST* mutant showed no activation of *lrgA* at any point in growth. The *lytST* overexpressing strain showed slightly elevated fluorescence prior to stationary phase and a large burst with increasing fluorescence when transitioning to, and remaining in, stationary phase. Therefore, we did not attempt to study *lrgAB* of individual cells carrying a *lytST* deletion. However, we did examine the effect of *lytST* overexpression under microfluidic flow.

**Figure 7:**
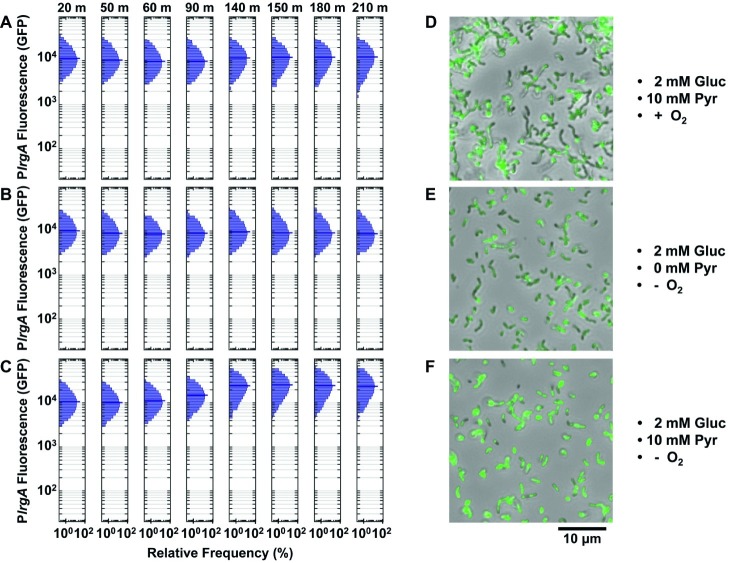
Effect of *lytST* on activation of a P*lrgA-gfp* reporter in flowing medium of fixed composition. The histograms show the green fluorescence of individual cells adhered within a microfluidic channel and subject to a steady flow of fresh, defined medium. Conditions are **(A)** aerobic medium containing 2 mM glucose/10 mM pyruvate; **(B)** anoxic medium containing 2 mM glucose (no added pyruvate); **(C)** anoxic medium containing 2 mM glucose/10 mM pyruvate. In **(D–F)**, microscopy images of the *lytST*-overexpressing strain with P*lrgA*-*gfp* (SAB163/P*lrgA-gfp*) in phase-contrast (gray scale) are overlaid with GFP fluorescence (green) images collected at 150 min. Five individual images were collected and analyzed for each experimental condition shown.

We loaded a *lytST* overexpression strain harboring the P*lrgA-gfp* reporter into microfluidic channels as above. Figure 7A shows the response of cells that were provided aerobic (air-equilibrated) defined medium containing 2 mM glucose and 10 mM pyruvate. Expression of *lrgA* remained constant throughout the experiment but with a median fluorescence nearly 1.7- to 3.4-fold greater than wild type cells in a similar but deoxygenated medium (in Figures 5B,D). Therefore, the overexpression of *lytST* bypasses the *lrgA* requirement for deoxygenation.

We tested whether pyruvate was needed to activate the *lrgA* reporter in the *lytST* overexpressing strain. Figure 7B shows that *lrgA* activated in anoxic medium containing 2 mM glucose lacking added pyruvate. Robust expression of *lrgA* was nearly identical to Figure 7A. We also tested activation of *lrgA* in anoxic medium with 2 mM glucose and 10 mM added pyruvate (Figure 7C), which were necessary to activate *lrgA* in the UA159 background. After 90–140 min of flow, expression of *lrgA* increased to about 2-fold greater than in Figures 7A,B. Figures 7D–F show cells with activated *lrgA* reporters (green). These data show that although *lytST* overexpression alleviates the requirement for anoxic conditions in activating *lrgA*, it does not entirely eliminate sensitivity to external pyruvate.

Finally, the population distribution of individual cell fluorescence in the *lytST* overexpression strain was observed to be slightly narrower than in the UA159 background, Figures 5B,D.

## Discussion

The *cidAB* and *lrgAB* operons were first identified as a putative holin-antiholin system in *Staphylococcus aureus*, with gene products that control extracellular murein hydrolase activity (Brunskill and Bayles, 1996; Groicher et al., 2000; Rice et al., 2003; Rice and Bayles, 2003). The *S. aureus lrgAB* operon is activated differentially through the growth curve, with the largest number of RNA transcripts detected during the transition from exponential to stationary phase (Brunskill and Bayles, 1996; Groicher et al., 2000). Studies of *S. mutans lrgAB* have found generally similar patterns of expression (Ahn et al., 2010; Kim et al., 2019), although these transcriptional studies have not yielded a precise determination of the environmental cues that control the operon. By combining a fluorescent gene reporter for *lrgA* with single-cell observations and microfluidic control of growth media conditions, we obtained a more detailed understanding of the environmental signals that trigger *lrgAB* in early stationary phase.

Several previous studies (Ahn et al., 2010; Kim et al., 2019) showed that higher glucose concentrations suppress *lrgAB* expression, and a recent study found a binding site for the catabolite repressor protein CcpA on the *lrgA* promoter region (Kim et al., 2019). The fact that *lrgA*, like many other virulence-linked genes in *S. mutans*, is regulated by catabolite repression *via* CcpA (Abranches et al., 2008) could potentially explain the burst of *lrgA* expression at the end of exponential growth. However, our data imply that an additional input also suppresses *lrgA* during the exponential phase. Deletion of *ccpA* did not affect the timing of the expression burst (Figure 6), although it did increase the level of that expression by as much as two fold (Ahn and Rice, 2016).

Molecular oxygen was found to exert more decisive control over the *lrgA* burst. No combination of glucose/pyruvate concentrations was found to activate *lrgA* in cells that grew in a continuous flow of fresh, defined medium, unless that medium was rigorously deoxygenated. In deoxygenated medium, robust *lrgA* expression occurred even though the composition of the medium (FMC medium containing added pyruvate) was otherwise compatible with normal, exponential growth. This finding suggests that the population-wide, tightly synchronized burst of *lrgA* expression observed in static, bulk cultures at stationary phase is not triggered by an internal state of the bacteria or by accumulation of pyruvate or depletion of nutrients from the media, but rather by the coincidence of oxygen depletion with availability of pyruvate. This can be seen in Figures 5C,E where both channels failed to activate *lrgA* in the absence of pyruvate even though the medium was anoxic. The exhaustion of oxygen, in combination with the presence of pyruvate, is an environmental condition that presumably occurs at a well-defined time point during the growth curve and can trigger P*lrgA* abruptly, unlike more gradual changes like the accumulation of a waste product or a quorum sensing signal.

As a facultative anaerobe, *S. mutans* undergoes fermentation in the absence of oxygen, converting pyruvate to lactic acid as one of its end products (Abbe et al., 1982). In the presence of oxygen, *S. mutans* is capable of aerobic respiration (Thomas and Pera, 1983), and so, the effect of oxygen availability on *lrgAB* regulation in static, aerobic cultures was previously investigated by microarray analysis (Ahn et al., 2007). Higher total *lrgA* RNA was found in aerobic cultures than in anaerobic cultures during mid-exponential phase (optical density of 0.4 at 600 nm) (Ahn et al., 2007). A follow-up study similarly found more pronounced *lrgAB* expression at stationary phase under aerobic conditions than under low-oxygen conditions (Ahn et al., 2012). Our present study differs from these prior studies in some key respects. One is that our use of a fluorescent reporter provides higher temporal resolution for detecting and probing the burst of *lrgAB* activity that occurs at stationary phase, which transcriptional studies may have missed. It is possible that metabolic changes in response to oxygen concentration have affected RNA stability in prior studies (Vargas-Blanco et al., 2019). Further, the low-oxygen conditions in Ahn et al. (2007) and Ahn et al. (2012) were less well defined than in the present study. For example, the low-oxygen condition in Ahn et al. (2012) consisted of growth in 5% CO_2_, which is not equivalent to the more anaerobic condition achieved here through the use of an enzymatic oxygen scavenger. Our data clearly show that tight control over oxygen concentration, in addition to high time resolution, are both necessary in the strong upregulation of *lrgA* early in stationary phase.

The mechanism by which oxygen represses *lrgAB* is not known, although the VicRK two component system is a potential candidate that has been shown to influence *lrgAB* expression (Supplementary Figure S2; Ahn and Rice, 2016). VicRK has also been linked to oxidative stress tolerance in *S. mutans* (Ahn and Burne, 2007; Deng et al., 2007; Senadheera et al., 2007) and VicK is regarded as a potential sensor of oxygen or redox conditions through its PAS domain (Taylor and Zhulin, 1999). A transcriptional study found that deletion of *vicK* led to moderate increase in early exponential phase expression of *lrgA*, but a nearly 100-fold decrease in late exponential phase (Ahn and Rice, 2016). In contrast, our bulk, fluorescence study did not show a decrease in *lrgA* activity at the transition into stationary phase (Supplementary Figure S2). Since reaching stationary phase is critical for the activation of *lrgA*, a transcriptional study may have been inadequate to capture *lrgA* activation in (Ahn and Rice, 2016). Although we did not investigate *vicK* effects at the individual cell level, our bulk culture data provide some support for the VicRK system as an oxygen sensing system that modulates *lrgAB* activity. The deletion and overexpression of *vicK* did not make a substantial difference in the behavior of the P*lrgA* reporter during anaerobic growth, but the *vicK* deletion strain showed modest P*lrgA* activity following aerobic growth, unlike either the *vicK* overexpression strain or wild type background. These data suggest that *vicRK* does not play a large role under anaerobic conditions, but that in the presence of molecular oxygen, it provides a mechanism that suppresses the onset of *lrgAB* expression.

LytST has also been identified as a potential intermediate between molecular oxygen and *lrgA* (Ahn et al., 2010, 2012). However, LytST homologs in other organisms have more recently been identified as sensors of extracellular pyruvate. In *Escherichia coli*, two-component systems of the LytS/LytTR family have been identified as receptors for external pyruvate (Behr et al., 2014, 2017), and a LytST-regulated system is triggered by extracellular pyruvate (Vilhena et al., 2018). In a recent study, *E. coli* in a viable but non-culturable state were rescued by pyruvate (Vilhena et al., 2019). In *B. subtilis*, both *lytST* and the *lrgA* homologs, *ysbA* and *pftA*, were shown to be essential for pyruvate utilization (van den Esker et al., 2017a). As in *S. mutans*, *B. subtilis ysbA* activates at the onset of stationary phase and decreases its expression with increasing initial glucose concentrations due to regulation by CcpA (Charbonnier et al., 2017; van den Esker et al., 2017a). The *ysbAB* (or *pftAB*) operon is induced by LytST in the presence of extracellular pyruvate (Charbonnier et al., 2017). That study reported that PftA and PftB form a hetero-oligomer that functions as a pyruvate-specific facilitated transporter and, together with LytST, help to adapt to a changing environment when the preferred carbon sources have been exhausted (Charbonnier et al., 2017).

Certainly, the LytST system is a key regulatory input to *lrgAB* expression in *S. mutans*, as deletion of *lytST* was previously shown to prevent stationary phase expression of *lrgAB* (Supplementary Figure S4; Ahn et al., 2010). In our studies, a *lytST* overexpressing strain readily activated *lrgA*, even in the absence of pyruvate and in media that were not thoroughly deoxygenated. Overexpression of *lytST* eliminated the bursting character of *lrgA* expression and caused instead generally robust expression under aerobic and pyruvate-absent conditions, where *lrgA* expression was absent in the wild type. These data indicate that *lytST* is not only required for activation of *lrgA*, but that it can overpower the repression signals due to molecular oxygen. Interaction of LytST with the *cre1* site previously suggested that LytST may inhibit the action of CcpA and therefore partially bypass catabolite repression as well (Kim et al., 2019).

The brief duration of strong *lrgA* expression at stationary phase may offer an intriguing clue to the mechanism of regulation, as it suggests a self-limiting behavior. In the *B. subtilis* study above, induction of the *lrgAB* homolog *ysbAB* (*pftAB*) increased as pyruvate increased up to 1 mM but was also inhibited *via* LytST under excess pyruvate conditions (Charbonnier et al., 2017), suggesting that an influx of pyruvate led to inhibition. One may speculate that if expression of *S. mutans lrgA* triggers a pyruvate influx that suppresses further *lrgA* expression, then the temporal profile of *lrgA* activity in response to extracellular pyruvate would appear as a rapid burst as is observed here. In that case, if pyruvate can also enter the cell by another pathway (unrelated to LrgAB and LytST), then very-high concentrations of extracellular pyruvate would be expected to suppress *lrgAB* activity, as is observed.

**Figure 8:**
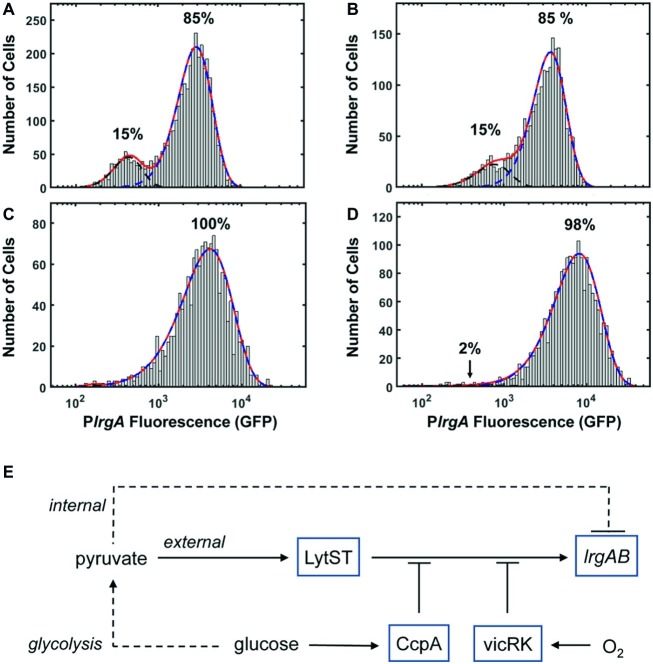
P*lrgA-gfp* reporter activity of individual cells fit to two gamma distributions representing the activated (dashed blue) and inactivated (dashed black) populations. Solid red line is the sum of both distributions. **(A)** 7 h sample from Figure 4C; **(B)** 8 h sample from Figure 4C; **(C)** 210 min sample from Figure 5B; **(D)** 210 min sample from Figure 5D. Five individual images were collected and analyzed for each experimental condition shown. **(E)** Schematic for control of *S. mutans lrgAB* by glucose, molecular oxygen, and external and internal pyruvate signals, *via* LytST and vicRK.

It is an interesting property of *lrgAB* that its activation (and subsequent deactivation) in a bulk culture is tightly synchronized temporally in the population, and yet the level of expression (as indicated by GFP concentration) varies between cells. Although some of the cell-to-cell heterogeneity seen in *S. mutans* fluorescent protein expression can probably be attributed to the use of plasmid-based reporters (Shields et al., 2019), the heterogeneity we observe in *lrgA* expression cannot be due entirely to the plasmid reporter. When cells drawn from a static culture activate *lrgA*, the population distribution in fluorescence is broad with a strongly bimodal character (Figure 4C). This bimodality is highlighted in Figures 8A,B, which represent each of the *lrgA-*active, single-cell histograms as the sum of two gamma probability distributions [The gamma distribution is characteristic of stochastic gene expression (Friedman et al., 2006)]. The relative areas under the two distributions indicate that roughly 85% of cells are *lrgA-*active (high fluorescence) at both 7 and 8 h. By contrast, *lrgA* expression under microfluidic flow (Figures 5B,D) lacks this bimodal character, producing virtually unimodal histograms (≥ 98% *lrgA-*active) in the same mathematical representation (Figures 8C,D). This finding indicates that, when environment conditions are sufficiently uniform as in the microfluidic study, a robust and generally similar level of *lrgA* expression is observed population wide. Therefore, local differences or gradients in key parameters such as pyruvate, oxygen, or glucose may explain some of the heterogeneity that was observed in our static culture studies and also in the individual cell expression of the *lrgA* homolog *ysbA* in *B. subtilis* (van den Esker et al., 2017a). The oral biofilm presents *S. mutans* with a highly heterogeneous environment (Stewart and Franklin, 2008), including variable oxygen concentrations. Our data suggest that this heterogeneity could induce very diverse levels of *lrgAB* activation in a biofilm population, leading to a useful division in response. Cells deeper within the biofilm would encounter less oxygen while presumably receiving pyruvate *via* diffusion from cells nearer the surface layer and would then be significantly more likely to activate the *lrgAB* mechanism and utilize the pyruvate source.

The presence of heterogeneity without bimodality in our microfluidic data also implies that *lrgAB* is regulated in an open-loop mechanism, without benefit of the positive transcriptional feedback that is typically associated with bimodality in gene expression (Dubnau and Losick, 2006). Rather, a mechanism of activation by LytST followed by negative feedback *via* intracellular pyruvate, as hypothesized above (Figure 8E), may be sufficient to control *lrgAB*, as it allows both an on-switch and an off-switch. Therefore, our data substantially revise the proposed model (van den Esker et al., 2017b) for *lrgAB* regulation in *S. mutans* by showing that intracellular and extracellular pyruvate modulate *lrgAB* expression, most likely through LytST, and that oxygen also plays a very strong repressing role that is likely facilitated by VicRK. Taken together, our model combines the three parameters studied here to illustrate their role in *lrgAB* activation. We note that the histogram of single-cell fluorescence is markedly narrower for the *lytST* overexpressing strain (Figure 7A) than for the wild type background (Figure 5D), suggesting that *lytST* overexpression is a strong enough stimulus that it brings *lrgA* expression closer to saturation and reduces the heterogeneity that is normally present.

Finally, our study has not identified a pathway by which *cidAB* modulates *lrgAB* expression. These two operons exhibit a complex pattern of transcriptional cross regulation that is growth-phase dependent, indicative of interactions between different gene products within both operons. It is likely not as simple as mutual repression (Ahn and Rice, 2016). Future studies of *cidAB* activation may begin to shed light on how the two operons interact.

## Data Availability Statement

The datasets generated for this study are available on request to the corresponding author.

## Author Contributions

II designed and performed experiments, analyzed and interpreted data, and wrote the manuscript. SH designed experiments, analyzed and interpreted data, and wrote the manuscript. KR and S-JA interpreted data and edited the manuscript. All authors gave final approval to the manuscript and agree to be accountable for all aspects of the work.

### Conflict of Interest

The authors declare that the research was conducted in the absence of any commercial or financial relationships that could be construed as a potential conflict of interest.
