# Supplementary material for: Environmental Triggers of lrgA Expression in Streptococcus mutans
Source: Front Microbiol. 2020 Jan 28;11:18. doi: 10.3389/fmicb.2020.00018 (PMC6997555; doi:10.3389/fmicb.2020.00018)
Supplement: Supplementary file 1 [file Data_Sheet_1.PDF]

Supplemental Material

List of supplementary information and figures.

- Figure S1: Effect of anaerobic versus aerobic growth on the step increase in *lrgA* activity at stationary phase in static cultures.
- Figure S2: Aerobic and anaerobic growth curves and green fluorescence of P*lrgA-gfp* reporter and non-reporter strains carrying a deletion of *vicK*, a *vicK* overexpressing plasmid or a UA159 background.
- Figure S3: Microfluidic study of the effect of O_2, glucose and pyruvate on non-reporting UA159, a P*lrgA-gfp* reporting strain, and strain carrying a constitutive P*ldh-gfp* reporter.
- Figure S4: The burst of *lrgA* expression at stationary phase of static cultures grown anaerobically carrying either a *lytST* deletion mutant or a *lytST* overexpression plasmid.

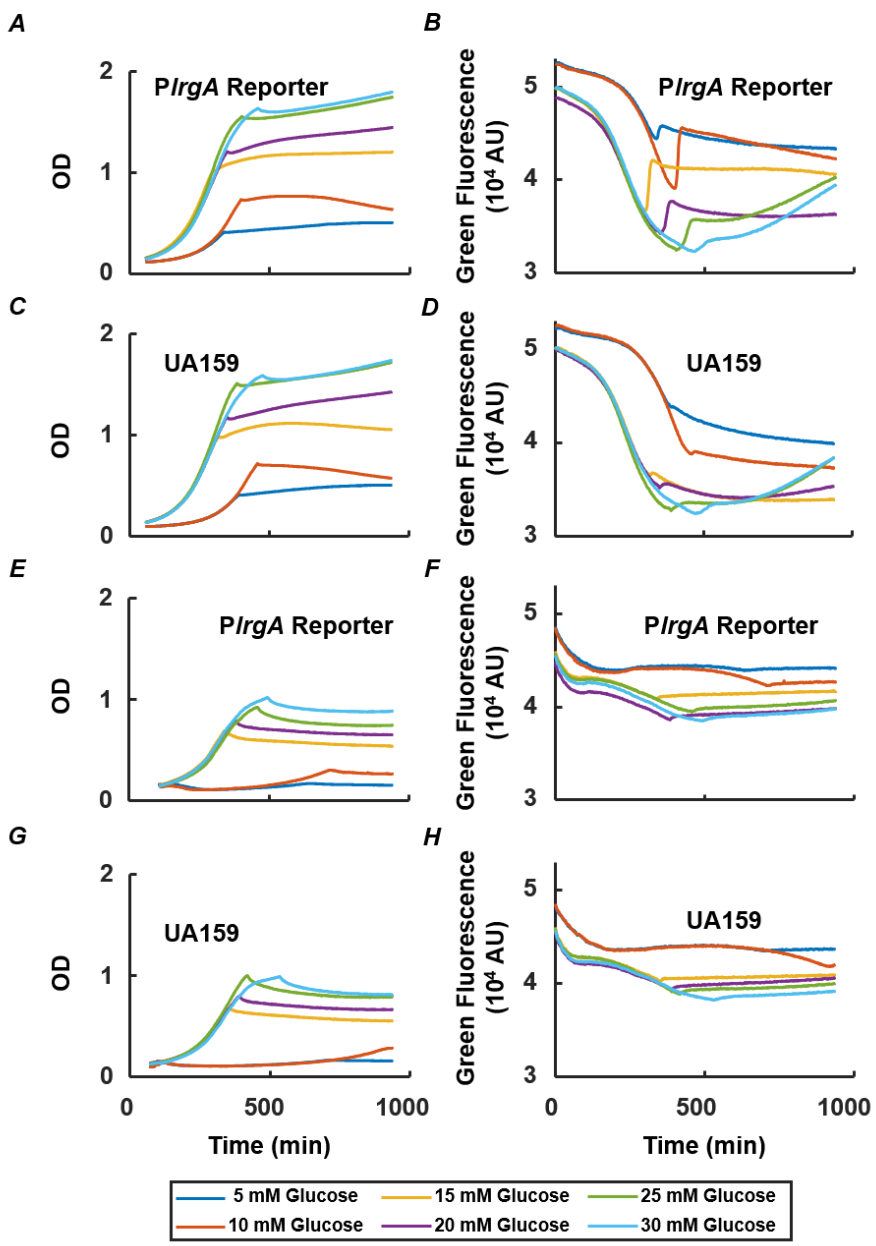

Figure S1: Effect of anaerobic versus aerobic growth on the step increase in *lrgA* activity at stationary phase in static cultures. (**A**) Optical density and (**B**) green fluorescence of anaerobically grown P*lrgA-gfp*; (**C**) optical density and (**D**) green fluorescence (background) of non-reporting, UA159 background strain; (**E**) optical density and (**F**) fluorescence of aerobically grown P*lrgA-gfp* strain; (**G**) optical density and (**H**) fluorescence of aerobically grown non-reporter UA159 background strain. All strains were grown in defined medium (FMC) containing initial glucose concentrations as indicated. The data shown in each panel represent one of three independent samples that were measured simultaneously.

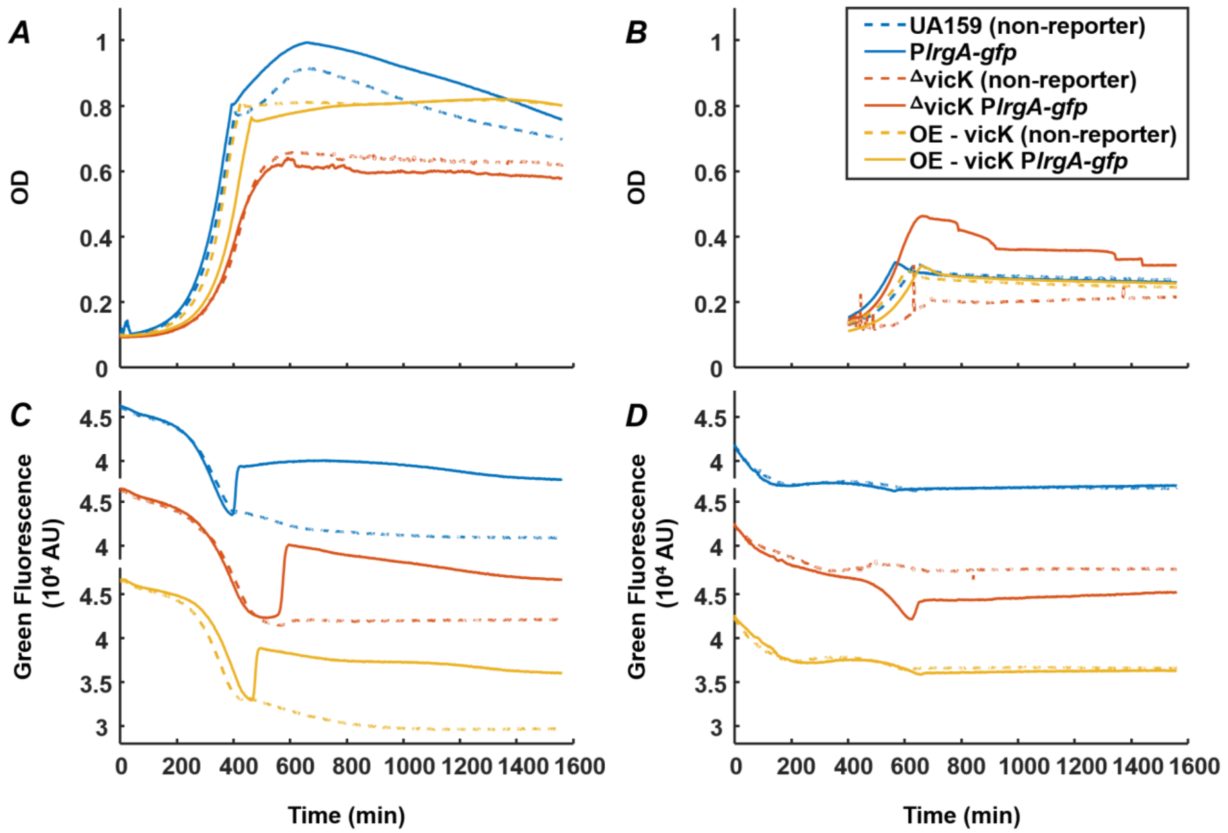

Figure S2: Growth curves and green fluorescence of P*lrgA-gfp* reporter (solid curves) and non-reporter (dashed curves) strains carrying a deletion of *vicK* (orange curves), a *vicK* overexpressing (OE) plasmid (yellow curves) or a UA159 background (blue curves). (**A**) Optical density and (**C**) green fluorescence of cells grown anaerobically; (**B**) Optical density and (**D**) green fluorescence of cells grown aerobically. All cells were grown in defined medium containing 10 mM initial glucose.

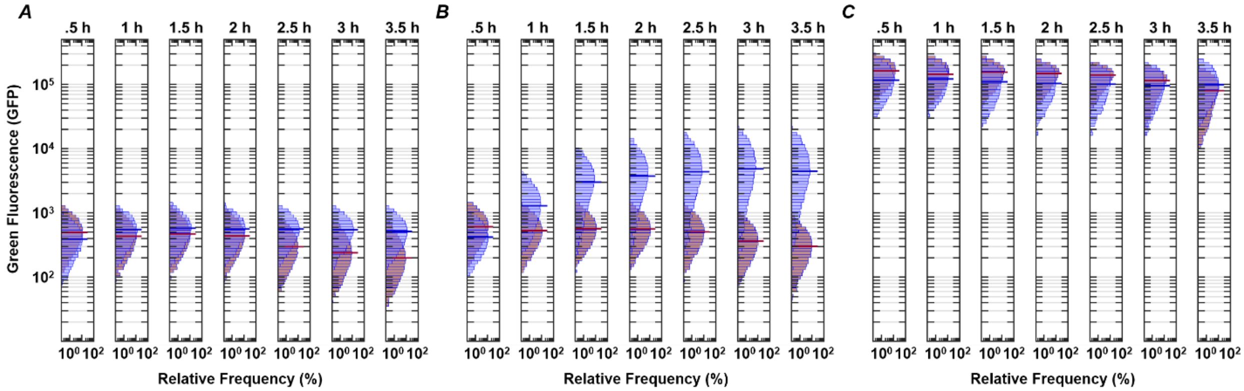

Figure S3: Microfluidic study of the effect of O_2, glucose and pyruvate on (**A**) non-reporting UA159, (**B**) a P*lrgA-gfp* reporting strain, and (**C**) strain carrying a constitutive P*ldh-gfp* reporter. All cells were adhered in microfluidic channels and provided a continuous flow of fresh, defined medium containing 5 mM glucose, 10 mM pyruvate. Medium was either aerobic (red) or anoxic (blue). The length of each horizontal bar indicates the percentage of cells that fluoresced at the indicated level. The heavy horizonal line in each histogram indicates the median fluorescence of the population. Five individual images were collected and analyzed for each experimental condition shown.

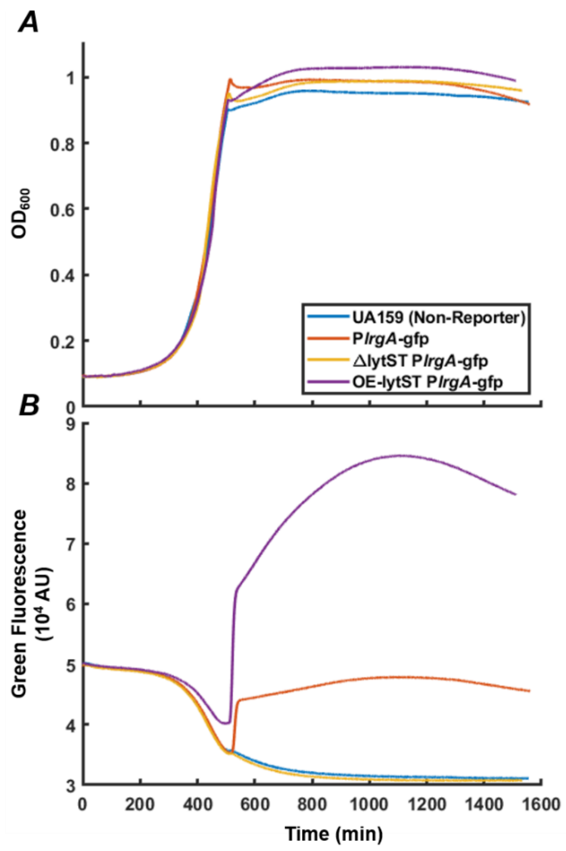

Figure S4: The burst of *lrgA* expression at stationary phase of static cultures grown anaerobically in defined media containing 10 mM initial glucose. (**A**) Growth and (**B**) green fluorescence of PlrgA-gfp reporting strains including a *lytST* deletion mutant and a *lytST* overexpressing strain.
